# P-1741. Incident Coccidiomycosis at Stanford Health Care

**DOI:** 10.1093/ofid/ofaf695.1912

**Published:** 2026-01-11

**Authors:** Markus Guerrero, Rebecca Y Linfield, Honor Magon, Daisuke Furukawa

**Affiliations:** Stanford Health Care, Stanford, California; Stanford University, Stanford, California; Atropos Health, New York, New York; Stanford University, Stanford, California

## Abstract

**Background:**

Cases of coccidioidomycosis are increasing dramatically, rising in California 8-fold from 1,491 cases in 2001 to 12,637 cases in 2024. Climate change is thought to contribute due to wide hydroclimatic swings. There is limited literature on healthcare utilization for patients with coccidioidomycosis, with one recent study citing that 30% of patients with disseminated disease ultimately required hospitalization during follow up. We sought to characterize the emergency room (ER) and hospitalizations within the first year of diagnosis at Stanford Health Care (SHC).

**Methods:**

We assessed health care utilization among SHC patients with an index diagnosis of coccidiomycosis (ICD-10 code B38) from 2016 to 2023, focusing on Emergency Department (ED) visits and inpatient admissions for the first year following diagnosis.

**Results:**

Our institution saw 87-129 (median 95, IQR 89.8-105) individuals annually with incident coccidioidomycosis. The annual rates of incident diagnosis varied without a discernible trend. The overall healthcare utilization of these patients was elevated. Total all-cause ED visits ranged from 11.5-18.2% (median 13.8%, IQR 13.1-17.3%). All-cause inpatient admissions ranged from 21.8-46.6% (median 35.0%, IQR 34.0-37.5%).

**Conclusion:**

As a tertiary referral center, incident cases of coccidioidomycosis stayed fairly constant over the study period. We identified significant healthcare utilization among these patients. It is imperative to ensure sustained expertise for inpatient and follow-up care, including infectious disease, pulmonology, neurology, neurosurgery, and orthopedic surgery as required. Further work will analyze healthcare utilization based on whether patients have disseminated disease.

**Disclosures:**

All Authors: No reported disclosures

